# Palmitoylation in Renal Physiology and Pathology

**DOI:** 10.3390/biom16030473

**Published:** 2026-03-22

**Authors:** Jingru Ma, Zhen Zhang, Jiaqi Guo, Hu Cai, Jian Yao, Dahai Yang, Huiyuan Zhu, Haijing Liu, Changhe Wang, Hongbo Xu

**Affiliations:** 1 Shaanxi Collaborative Innovation Center of Chinese Medicinal Resources Industrialization, Shaanxi University of Chinese Medicine, Xianyang 712046, China; 224100012974@email.sntcm.edu.cn (J.M.); zhen@sntcm.edu.cn (Z.Z.);; 2 Shaanxi Medical Devices Quality Testing Institute, Xixian New Area, Xianyang 712046, China; 3 Division of Molecular Signaling, Department of the Advanced Biomedical Research, Interdisciplinary Graduate School of Medicine, University of Yamanashi, Chuo 409-3898, Japan; 4 Shaanxi Center for Technical Review of Pharmaceutical Products, 56 Gaoxin 6th Road, Yanta District, Xi’an 710065, China; 5 Shaanxi Institute for Food and Drug Control, High-Tech Zone, Xi’an 710075, China

**Keywords:** palmitoylation, kidney, post-translational modification, palmitoylation detection

## Abstract

Palmitoylation is a critical post-translational modification that involves the covalent binding of palmitic acid to cysteine residues within proteins. It is widely recognized that palmitoylation plays a significant role in regulating protein membrane localization, stability, and interactions. The kidney plays a key role in maintaining fluid homeostasis and excreting metabolic waste, and its normal function relies on the precise regulation of protein function. Emerging evidence reveals the crucial role of palmitoylation in renal physiological and pathological processes. However, the intricate pathways and molecular regulators in the kidney that are involved in palmitoylation remain insufficiently elucidated. This review summarizes the role and possible underlying physiological and pathological mechanism of palmitoylation in the kidney, including enzymes and inhibitors that regulate palmitoylation, the signaling pathways involved, target proteins involved in palmitoylation, and specific modification sites. Moreover, we focus on detection techniques and corresponding research strategies for palmitoylation. This review can also serve as a practical reference to improve the understanding of palmitoylation and the treatment of kidney-related diseases.

## 1. Introduction

**Figure 1:**
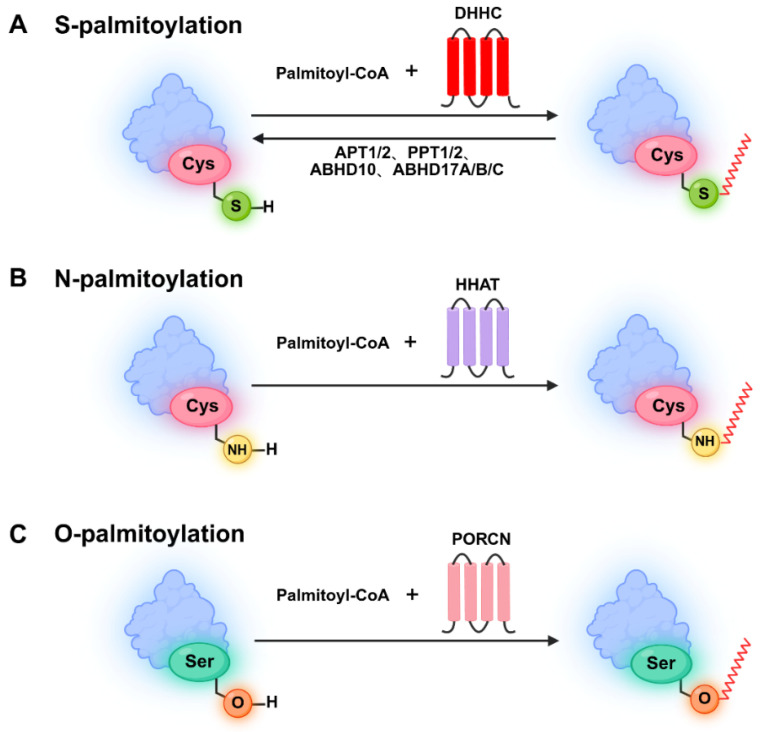
Reaction mechanisms involved in palmitoylation formation. Palmitoylation occurs via (**A**) S-palmitoylation on cysteine thiols (Cys-SH); (**B**) N-palmitoylation on cysteine amino groups (Cys-NH_2_); (**C**) O-palmitoylation on serine hydroxyl groups (Ser-OH).

Palmitoylation is a post-translational modification in which palmitoyltransferase covalently links 16-carbon palmitate to protein cysteine residues, which regulates protein membrane localization, stability, and interactions [1]. Palmitoylation can be classified into S-palmitoylation, N-palmitoylation, and O-palmitoylation based on the specific cysteine modification site [2]. Currently, S-palmitoylation, a reversible and dynamic lipid modification process that links long-chain fatty acids to cysteine residues through labile thioester linkages [3], is the most studied form of palmitoylation. N-palmitoylation refers to the covalent attachment of a palmitoyl group via an amide bond to the alpha-amino group of N-terminal glycine or cysteine, as well as to the side chain amino group of lysine. A prominent example is the modification of Sonic hedgehog (SHH) at its N-terminal cysteine, which is specifically catalyzed by Hedgehog acyltransferase (HHAT) [4,5,6]. O-palmitoylation refers to the esterification of palmitic acid with the hydroxyl group of serine or threonine residues (Figure 1).

Recently, research on protein palmitoylation has increased. A PubMed database search with the keywords “palmitoylation” and “kidney” revealed 493 relevant publications. Bibliometric analysis reveals a fluctuating upward trend within this field since 1981, peaking at 27 publications in 2022. Emerging evidence from these studies reveals the crucial roles of palmitoylation in renal physiological and pathological processes.

**Figure 2:**
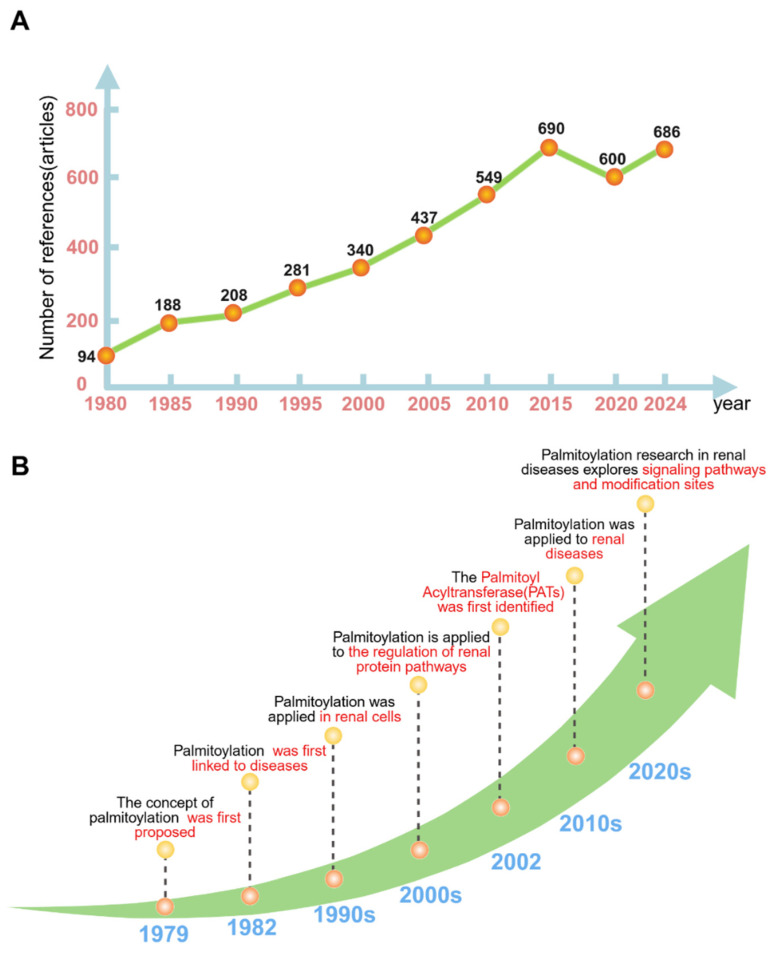
Analysis of the development history of palmitoylation. (**A**) Annual statistics of the number of papers published every five years during the period from 1980 to 2024; (**B**) research progress on palmitoylation in kidney diseases since its first report in 1979.

The concept of palmitoylation was first proposed by Schmidt et al. in 1979 [7]. In 1982, Sefton et al. provided the first evidence that the Ras protein binds lipids, specifically palmitate, and reported that the Ras protein underwent palmitoylation [8]. Given that Ras is a known oncoprotein, this discovery forged the initial connection between palmitoylation and diseases, particularly cancer, laying a crucial foundation for subsequent investigations into the role of palmitoylation in disease pathogenesis. In the 1990s, the role of palmitoylation in kidney cells began to emerge, which focused primarily on the effects of palmitoylation on protein localization and fundamental functions within these cells. In 2002, Roth et al. employed a genetic screening approach in yeast targeting the palmitoylation-dependent Ras2 protein, thereby achieving the first ever identification of the palmitoyl acyltransferase (PAT) family [9]. The PAT family is composed of 23 distinct proteins, each containing a highly conserved Asp-His-His-Cys (DHHC) motif essential for catalytic activity. Consequently, these proteins were also designated zinc-finger DHHC-containing (ZDHHC) proteins [10]. In the 2000s, the study of palmitoylation focused on pathway regulation, which revealed that palmitoylation is involved in various biological processes, such as Wnt signaling, cholesterol metabolism, and the membrane trafficking of proteins specific to the kidney. The rapid development of genomics and proteomics has provided powerful tools to systematically screen for key palmitoyltransferases and verify their downstream substrate proteins. Breakthroughs in high-throughput mass spectrometry have enabled the identification of hundreds of palmitoylated proteins in the kidney. Notably, techniques such as acyl-resin-assisted capture(acyl-RAC) combined with LC-MS/MS have been used to characterize sodium channel proteins, including ENaC, and to achieve the large-scale discovery and precise mapping of palmitoylation sites. In the 2010s, research on palmitoylation extended to the field of kidney diseases. This advancement established an association between hereditary kidney disorders (e.g., Bartter syndrome) and metabolic kidney diseases (e.g., diabetic kidney disease). In the 2020s, research on palmitoylation began to reveal specific modification sites on its target proteins (Figure 2).

These studies have not only shed light on the crucial roles that palmitoylation plays in renal physiological and pathological processes but also presented novel diagnostic approaches and potential therapeutic strategies for kidney diseases. Consequently, we have conducted a comprehensive summary of the palmitoylation research pertaining to renal physiology and pathology.

## 2. Reaction and Regulatory Mechanisms of Palmitoylation

### 2.1. Reaction of Palmitoylation

**Figure 3:**
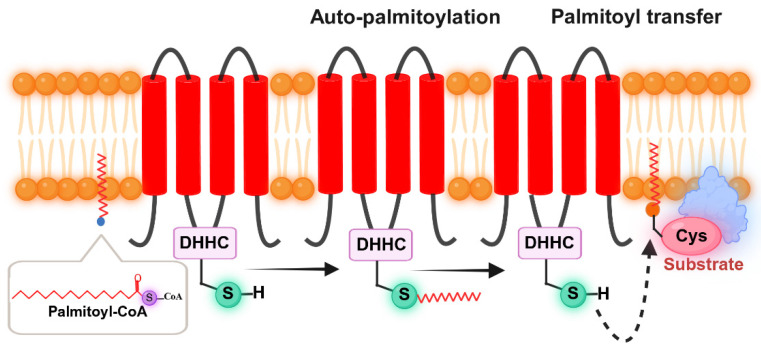
Process of palmitoylation modification. It mainly consists of two steps: the automatic palmitoylation reaction and the palmitoyl transfer reaction.

Palmitoylation is a pivotal protein post-translational modification, the process of which predominantly occurs in two essential steps. The first step involves the autoacylation reaction. During this process, palmitic acid reacts with coenzyme A (CoA) to yield palmitoyl-CoA. Subsequently, the generated palmitoyl-CoA binds to a cysteine residue located within a palmitoyl acyltransferase belonging to the DHHC family, which leads to the formation of an acyl-enzyme intermediate and the concurrent release of CoA-SH. The second step involves the acyl transfer reaction. In this reaction, the DHHC enzyme catalyzes the transfer of the palmitoyl group onto a cysteine residue of the substrate protein. This transfer ultimately leads to the completion of the palmitoylation modification (Figure 3). Furthermore, S-palmitoylation constitutes the major form of palmitoylation and exhibits reversibility. Specifically, depalmitoyltransferase can act on the protein and then remove palmitic acid from the cysteine residue and initiate the depalmitoylation process. This reversibility further shows how palmitoylation can be dynamically regulated.

### 2.2. Regulatory Mechanisms of Palmitoylation

#### 2.2.1. Palmitoyl Acyltransferases

Palmitoyl acyltransferases are key enzymes that catalyze protein palmitoylation. The primary members of this group include the zinc finger DHHC-type (ZDHHC) protein family, along with Porcupine (Porcn), Hedgehog acyltransferase (Hhat), and other related enzymes [11]. Notably, the vast majority belong to the DHHC family. In mammals, 23 distinct ZDHHC enzymes (ZDHHC1–ZDHHC24, excluding ZDHHC10) have been identified as catalysts of palmitoylation [10]. Although a few of these (e.g., ZDHHC5, 20, 21) are localized primarily to the plasma membrane, most reside in the endoplasmic reticulum (ER) and Golgi apparatus [12].

ZDHHC family members are key regulators of cell polarity, signal transduction, and metabolic balance through their mediation of specific substrate palmitoylation. At the physiological level, ZDHHC5 and ZDHHC8 palmitoylate ankyrin-G to maintain lateral membrane polarity in MDCK cells [13]; ZDHHC21 regulates vascular function by palmitoylating eNOS [14]. In ion channel regulation, ZDHHC7 promotes the palmitoylation of Barttin and activates the ClC-K channel [15]. In lipid metabolism, ZDHHC7, ZDHHC17, and ZDHHC6/16 cooperatively palmitoylate diacylglycerol kinase ε (DGKε) to maintain phospholipid homeostasis [16].

Notably, palmitoyl acyltransferases exhibit functional diversity and complexity in renal pathological processes. In clear cell renal cell carcinoma (ccRCC), some ZDHHC members display overlapping or opposing roles, and bioinformatic analyses show that ZDHHC3, 6, 14, 15, 21, and 23 are downregulated, which suggests a potential common tumor suppressive function. In contrast, ZDHHC2, 9, 17, 18, 19, and 20 are overexpressed and exert oncogenic effects [17,18]. Mechanistically, ZDHHC2 activates the AKT-mTOR pathway through AGK palmitoylation to drive proliferation and drug resistance [19], whereas high expression of ZDHHC19 directly correlates with a poor prognosis [20]. ZDHHC3 exerts a tumor-suppressive effect by mediating S-palmitoylation of SLC9A2 to regulate apoptosis [18]. In septic injury, DHHC21 promotes α1AR palmitoylation, which in turn activates the α1AR-mediated ERK pathway and induces vasoconstriction [21]. Furthermore, ZDHHC1 palmitoylates the human norepinephrine transporter (hNET) and promotes its transport capacity [22]. Notably, in fibrotic processes, ZDHHC9 inhibits fibrosis via promoting the palmitoylation of β-catenin [23]. Conversely, ZDHHC18 exacerbates fibrosis progression by catalyzing HRAS palmitoylation [20]. These findings demonstrate that different members of the ZDHHC family, through specific substrate modification, assume synergistic or antagonistic functions in tumorigenesis, immune regulation, and fibrotic processes.

#### 2.2.2. Depalmitoyltransferases

Depalmitoyltransferases catalyze depalmitoylation reactions by hydrolyzing thioester bonds to remove palmitoyl groups from the cysteine residues of proteins. The depalmitoyltransferase family includes acyl-protein thioesterase 1 (APT1/LYPLA1), acyl-protein thioesterase 2 (APT2/LYPLA2), palmitoyl-protein thioesterase 1 and 2 (PPT1, PPT2), and α/β-hydrolase domain-containing proteins (ABHDs) [24]. Depalmitoyltransferases regulate diverse physiological and pathological processes by dynamically modifying substrate palmitoylation. For example, PPT1 modulates the palmitoylation state and lipid raft localization of the D1 dopamine receptor and affects renal sodium excretion and blood pressure regulation [25]. Dysfunction of PPT1 is closely associated with hypertension and oxidative stress-related disorders. Another study demonstrated that APT1 stabilizes β-catenin protein levels and promotes its nuclear translocation through β-catenin depalmitoylation, which consequently exacerbates renal fibrosis. In contrast, pharmacological inhibition or genetic ablation of APT1 significantly attenuates fibrotic progression to suggest that APT1 may serve as a therapeutic target [23]. Therefore, as key negative regulators in the palmitoylation cycle, depalmitoyltransferases govern the localization and function of specific substrate proteins by removing their palmitoylation.

#### 2.2.3. Palmitoylation Inhibitors

Palmitoylation inhibitors are compounds that target protein palmitoylation, primarily through the inhibition of palmitoyltransferase activity. Currently, only a limited number of DHHC inhibitors, such as 2-bromopalmitate (2-BP) [26], tunicamycin [27], and cerulenin [28], have been reported. Among these, 2-BP remains the most widely used. 2-BP operates through two distinct mechanisms [29]: (1) as a palmitate analog, it competes with endogenous palmitoyl-CoA and thereby interferes with substrate palmitoylation catalyzed by palmitoyl acyltransferases. (2) It covalently binds to the cysteine residue within the conserved DHHC catalytic motif of ZDHHC enzymes, directly disrupts their activity, and prevents palmitoyl group transfer to substrates.

2-BP has been used in multiple kidney-related studies. For instance, it inhibits the palmitoylation of Scr3, which reduces exosome secretion and causes its abnormal accumulation in the nucleus [30]. In addition, 2-BP blocks the palmitoylation of polycystin-1 (PC1), which results in the enrichment of PC1 in the plasma membrane and an increase in the expression of its carboxyl terminal fragment (CTF). In contrast, the coadministration of the depalmitoyltransferase inhibitors HDFP and palmstatin B reduces the expression of PC1-CTF by maintaining the palmitoylation of PC1 [31]. Interestingly, 2-BP exerts distinct regulatory effects on different DHHC family members. Specifically, 2-BP inhibits the activity of ZDHHC2 and blocks its palmitoylation of AGK, and thus prevents the translocation of AGK to the plasma membrane and the subsequent activation of the AKT-mTOR pathway [19]. 2-BP also reduces the palmitoylation of HRAS by inhibiting the expression or activity of ZDHHC18, and consequently decreases HRAS membrane localization [20]. Additionally, it has been reported that 2-BP disrupts mitochondrial homeostasis through interference with the interaction between STING and VDAC2 [32]. 2-BP blocks BgtR α7 subunit palmitoylation and BgtR expression in PC12 cells [33].

Together, these findings demonstrate that 2-BP inhibits the palmitoylation of substrate proteins. This action broadly affects protein localization, stability, and downstream signaling pathways.

### 2.3. Regulation of Signaling Pathways by Palmitoylation

#### 2.3.1. Wnt/β-Catenin Signaling Pathway

The Wnt/β-catenin signaling pathway is an evolutionarily highly conserved mechanism that plays a central regulatory role in physiological processes, including cell proliferation, differentiation, and stem cell self-renewal [34]. β-catenin is a key mediator of fibrotic signaling, and its activity is regulated by palmitoylation. Palmitoylation of β-catenin by DHHC9 promotes its polyubiquitination and subsequent proteasomal degradation, restricts its nuclear accumulation, and ameliorates kidney fibrosis. In contrast, APT1-mediated depalmitoylation removes this modification, stabilizes β-catenin, increases its abundance and nuclear translocation, and exacerbates kidney fibrosis [23].

#### 2.3.2. PI3K/AKT/mTOR Signaling Pathway

The PI3K/AKT/mTOR signaling pathway is a critically important intracellular cascade that profoundly regulates cellular growth, autophagy, programmed cell death, energy metabolism, and protein synthesis [35]. This pathway is initiated upstream by phosphatidylinositol 3-kinase (PI3K), which generates secondary messengers to activate protein kinase B (Akt). Akt then phosphorylates downstream substrates to modulate the activity of the mammalian target of rapamycin complexes 1 and 2 (mTORC1/2) [36]. Research has indicated that aberrant activation of the PI3K/AKT/mTOR pathway is observed in approximately 50% of human cancers, which establishes it as one of the most prevalent oncogenic mechanisms. Furthermore, its constitutive hyperactivation frequently underlies therapeutic resistance [37], a phenomenon exacerbated by palmitoylation, this hyperactivation promotes tumor cell proliferation, survival, and metastasis through facilitated Akt phosphorylation and enhances the stability and signaling fidelity of upstream PI3K and downstream mTOR complexes. Sun et al. demonstrated that ZDHHC2-mediated S-palmitoylation of AGK diminishes sunitinib sensitivity in RCC through a mechanism involving AGK translocation to the plasma membrane and subsequent activation of the PI3K-AKT-mTOR signaling axis [19]. Within this pathway, mTORC1, a key regulatory complex, phosphorylates specific residues on its downstream target S6K to activate ribosomal protein S6 kinase, which orchestrates protein synthesis. In RCC, pharmacological inhibition of mTORC1 signaling suppresses S6K activity to inhibit tumor cell proliferation and restrain oncogenesis [32].

#### 2.3.3. Hippo Signaling Pathway

The Hippo pathway was initially discovered in *Drosophila melanogaster* as a critical regulator of tissue growth and plays a crucial role in organismal development. It is fundamentally involved in a wide range of biological processes, including cell growth, differentiation, organ size control, and regeneration [38]. Notably, dysregulation of the Hippo pathway is closely linked to the pathogenesis of diverse diseases, particularly human cancers. Aberrant function of the Hippo pathway and its downstream effectors, the transcriptional coactivators YAP and TAZ, exerts deterministic influences on cancer cell phenotypes [39]. The palmitoylation of TEAD proteins, the primary transcription factor partners of YAP/TAZ, is essential for their stability and activity. This lipid modification occurs within a conserved palmitoylation pocket. The binding of inhibitors to this pocket disrupts TEAD auto-palmitoylation, impedes its interaction with YAP/TAZ, and consequently suppresses its transcriptional activity. A study characterized SWTX-143 [40], a YAP/TAZ-TEAD inhibitor that binds the palmitoylation pockets of all four TEAD isoforms. This compound selectively inhibits growth in NF2-mutant renal cancer cell lines, which blocks the transcriptional output of the Hippo pathway and promotes tumor regression.

#### 2.3.4. MEK/ERK Signaling Pathway

The MEK/ERK signaling pathway is a central component of the mitogen-activated protein kinase (MAPK) cascade and governs critical cellular processes, including proliferation, differentiation and apoptosis [41]. Aberrant activation of this pathway is closely associated with the pathogenesis of various diseases. During renal fibrosis, ZDHHC18 catalyzes HRAS palmitoylation, which leads to its activation. The activated HRAS then triggers the MEK/ERK signaling cascade, causing phosphorylation of both MEK and ERK [20]. These phosphorylated kinases subsequently phosphorylate and activate the transcription factor RREB1. Activated RREB1 binds to cis-regulatory elements on the *Snail* and *Has2* genes to promote their expression. As *Snail* and *Has2* encode key mediators of Epithelial-Mesenchymal Transition (EMT), their upregulation drives partial EMT in renal tubular epithelial cells. This process enhances the synthesis and secretion of extracellular matrix components, which accelerates renal fibrosis progression. Moreover, genetic ablation of ZDHHC18 suppresses HRAS activation, reduces MEK/ERK phosphorylation, inhibits downstream signaling, and ultimately ameliorates renal fibrogenesis.

## 3. Palmitoylated Proteins in the Kidney

**Figure 4:**
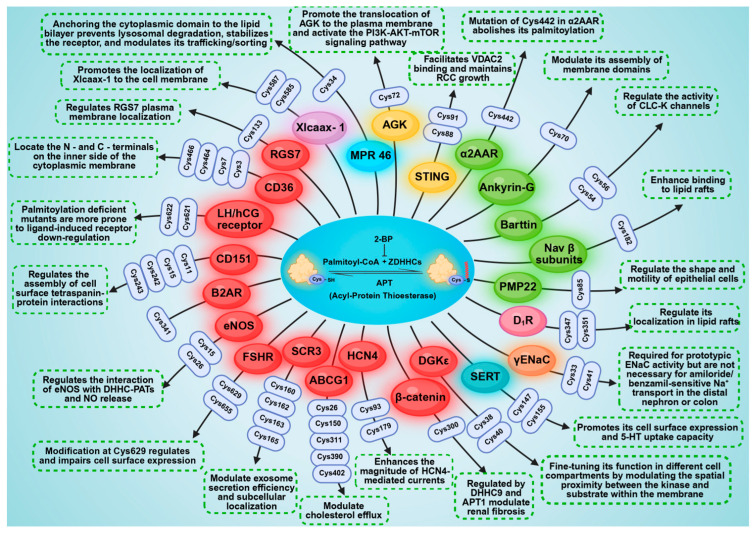
Schematic illustration of palmitoylated proteins in the kidney. Sites and functions of palmitoylated proteins (proteins marked with different colors indicate their presence in different cell types; red: HEK293 cells; green: MDCK cells; purple: XTC cells; blue: BHK21 cells; yellow: A498 cells; pink: RPTC cells; blue–green: AD293 cells).

To date, palmitoylation has been experimentally confirmed at more than 9000 specific sites on more than 3500 distinct proteins. Research on palmitoylation in renal tissues has increased substantially in recent years, underscoring its growing significance in both renal physiology and pathology. Considering these findings, we systematically summarize experimentally validated palmitoylation sites in renal cells and their associations with kidney diseases (Table 1), and summarized the sites and functions of palmitoylated proteins in the kidney (Figure 4). Besides, we also compile studies that report renal palmitoylation but do not identify specific sites (Table 2).

### 3.1. ENaC

The ENaC is a heterotrimeric complex consisting of α, β, and γ subunits that critically regulates renal water and sodium metabolism [75]. This channel specifically mediates sodium influx from the extracellular to the intracellular compartment and is essential for effective sodium reabsorption in the kidney. With respect to renal physiology, palmitoylation critically regulates ENaC activity, which influences sodium transport and reabsorption and maintains systemic sodium balance and normal physiological function [76]. The palmitoylation of the ENaC occurs predominantly at specific cysteine residues on the β and γ subunits and is catalyzed by DHHC7. Palmitoylation of the β subunit primarily enhances membrane localization and stability, whereas modification of the γ subunit directly modulates the probability of channel opening, enabling fine-tuning of sodium absorption. Nickerson et al. demonstrated that ablation of palmitoylation sites on the γ subunit significantly reduces ENaC activity in the kidney. Nevertheless, organisms may initiate compensatory mechanisms to counteract and partially offset this functional impairment of the ENaC, which preserves salt and fluid homeostasis [56]. In mutagenesis studies, the replacement of these palmitoylated cysteines with alanine enhances Na^+^-dependent self-inhibition, reduces the probability of channel opening, and does not alter ENaC surface stability or endocytosis [73]. These findings further corroborate the essential role of palmitoylation in the regulation of ENaC gating dynamics.

### 3.2. D_1_R

The dopamine D_1_ receptor (D_1_R) is a G protein-coupled receptor (GPCR) that is predominantly localized in renal tubules and vasculature [77]. It plays a pivotal role in renal physiology and pathology by regulating sodium excretion, renal blood flow, and blood pressure. Tiu et al. demonstrated that kidney-specific Drd1 silencing elevated blood pressure [25]. Although tubule-specific rescue with wild-type D_1_R restored normotension, the palmitoylation-deficient D_1_R 347A mutant failed to normalize blood pressure, indicating the indispensability of D_1_R palmitoylation for normal blood pressure regulation. Further investigations revealed that impaired D_1_R function, which results from either palmitoylation site mutation or lipid raft disruption, induces increased renal oxidative stress. This stress is accompanied by elevated urinary isoprostane levels and increased intracellular ROS production. These alterations not only damage renal cells but also may compromise renal function and thus contribute to the initiation and progression of kidney disease. Overall, the palmitoylation status of D_1_R is essential for preserving normal renal physiological function and mitigating renal pathogenesis.

### 3.3. Barttin

Barttin is an essential auxiliary subunit that modulates human ClC-K chloride channels, with expression primarily found in the kidney and inner ear. Palmitoylation can activate ClC-K/Barttin channels [15]. Specifically, proper palmitoylation of Barttin is essential for preserving renal ion homeostasis and promoting urinary concentration capacity. To investigate the functional impact of palmitoylation on Barttin, researchers generated a DHHC7 knockout mouse model, which exhibited significantly reduced palmitoylation levels of renal Barttin. Although these mice showed no overt renal dysfunction under normal dietary conditions, they manifested hyponatremia and mild metabolic alkalosis when they were fed a low-salt diet. These pathological features demonstrate high phenotypic recapitulation of human Bartter syndrome type IV. These findings further underscore the physiological significance of Barttin palmitoylation in renal function maintenance.

### 3.4. Cluster of Differentiation 36 (CD36)

CD36 is a transmembrane glycoprotein that requires precise subcellular localization to the plasma membrane and intracellular compartments for its physiological and pathological functions [78]. Its localization dynamics and functional activities are regulated by post-translational modifications, including palmitoylation. Mounting evidence underscores the essential function of CD36 in the pathogenesis of DKD [79]. For instance, studies in murine models of DKD have established that CD36 undergoes marked palmitoylation elevation in renal tissues compared with those in normal controls. This elevation coincided with profibrotic alterations, including increased tubular EMT and collagen deposition. These observations suggest that CD36 palmitoylation is involved in the initiation and progression of renal fibrosis in diabetic nephropathy. The underlying mechanism may be mediated by CD36 palmitoylation, which promotes EMT in renal tubular epithelial cells, consequently enhancing extracellular matrix protein secretion and ultimately culminating in renal fibrogenesis. These observations confirm that CD36 palmitoylation is involved in the initiation and progression of DKD; thus, it represents a potential therapeutic strategy for intervening in the progression of renal fibrosis [71].

### 3.5. ARL13b

ARL13b is a ciliary GTPase that undergoes palmitoylation in the murine kidney, a modification that is essential for maintaining its structural integrity and functionality within primary cilia—organelles critically involved in renal cellular signaling [80]. Consequently, the palmitoylation of ARL13b is a key determinant of proper ciliogenesis and ciliary function. Aberrant palmitoylation of ARL13b compromises the ciliary architecture and impairs its physiological activity [70], which subsequently disrupts signal transduction pathways in renal cells and thus may contribute to the pathogenesis of ciliopathy-associated renal disorders.

### 3.6. Cytoskeleton-Linking Membrane Protein 63 (CLIMP-63)

CLIMP-63 is an ER-resident protein that contributes critically to gentamicin-induced nephrotoxicity via its palmitoylation. Specifically, the binding of gentamicin induces the palmitoylation-dependent dimerization of CLIMP-63, which leads to the formation of dithiothreitol (DTT)-resistant dimers [69]. Knockdown of CLIMP-63 or inhibition of its palmitoylation reduces the sensitivity of renal proximal tubular cells to gentamicin. This intervention suppresses caspase-3 activity, enhances cell viability, and diminishes apoptosis. These findings elucidate the specific pathway through which CLIMP-63 palmitoylation mediates gentamicin-induced renal toxicity. Consequently, Targeting CLIMP-63 palmitoylation presents a potential novel approach to alleviate gentamicin-induced nephrotoxicity.

### 3.7. NaPi-II

TypeII sodium-phosphate cotransporters (NaPi-II), which belong to the solute carrier SLC34 family, are composed of three primary isoforms: NaPi-IIa, NaPi-IIb, and NaPi-IIc. These transporters are predominantly expressed in the proximal tubular epithelial cells of the kidney. The C-terminus of NaPi-IIb contains a conserved cysteine residue motif that undergoes palmitoylation [66]. Aberrant palmitoylation prevents NaPi-IIb from localizing properly to the apical membrane and disrupts normal phosphate transport. Renal dysregulation of this process can lead to either reduced phosphate reabsorption, resulting in significant phosphaturia and subsequent hypophosphatemia, or excessive reabsorption, which causes hyperphosphatemia. Both outcomes impair renal function and disrupt systemic phosphate homeostasis.

### 3.8. Podocin

Podocin is an essential protein for the function of the filtration barrier in the mammalian kidney and serves as a critical component of the multiprotein complex at the slit diaphragm of podocytes [80]. Mutations in predicted palmitoylation sites result in the loss of palmitoylation, which impairs the cholesterol-binding activity of podocin. Palmitoylation indirectly modulates mechanosensory processes that monitor the glomerular pressure and filtration rate, as it influences the interaction of podocin with cholesterol and its association with the TRPC6 channel [81]. Consequently, aberrant palmitoylation can disrupt the structure and function of this multiprotein complex. Such interference compromises the kidney’s capacity to monitor and regulate the glomerular pressure and filtration rate, which ultimately impairs renal filtration function.

## 4. The Role of Palmitoylation in Renal Pathophysiology

### 4.1. Chronic Kidney Disease (CKD)

**Figure 5:**
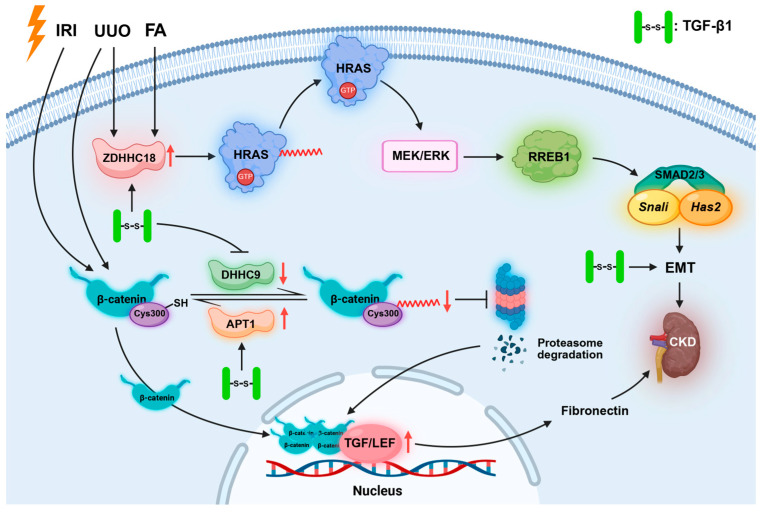
ZDHHC family enzymes promote the development of CKD through the palmitoylation of HRAS and β-catenin. The upward red arrow represents the upregulation of protein expression, the downward red arrow represents the downregulation of protein expression.

CKD is defined as persistent abnormalities in kidney structure or function that last longer than three months and feature a progressive decline in renal function, typically culminating in irreversible renal fibrosis. Patients with CKD frequently exhibit metabolic disturbances, which can disrupt normal palmitoylation processes and alter protein palmitoylation levels in renal cells. In both patients with CKD and UUO/IRI model mice, DHHC9 expression is downregulated, whereas APT1 expression is upregulated. Modulating the activity or expression of these enzymes shows promise for the therapeutic intervention in fibrosis. Specifically, the activation of DHHC9-mediated palmitoylation at Cys300 of β-catenin, or the inhibition of APT1 activity to prevent depalmitoylation, could promote the clearance of β-catenin and reduce its stability, and thereby effectively attenuate renal fibrosis [23]. Furthermore, Lu et al. reported a critical role for ZDHHC18 in CKD-associated renal fibrosis. ZDHHC18 is highly expressed in fibrotic kidneys from both patients with CKD and mouse models. It accelerates renal fibrosis progression by catalyzing HRAS palmitoylation and subsequently activating signaling pathways, including those involving MEK/ERK, RREB1, and SMAD2/3 [20]. This activation promotes partial EMT in renal tubular epithelial cells and leads to extracellular matrix accumulation and inflammatory responses (Figure 5). Therefore, the activation of DHHC9 to promote β-catenin palmitoylation or the inhibition of ZDHHC18 to prevent its catalysis of HRAS palmitoylation can inhibit renal fibrosis. TGF-β1 induces depalmitoylation of β-catenin through downregulation of DHHC9 or upregulation of APT1, and thereby promotes fibrosis. However, how TGF-β1 specifically regulates DHHC9 and APT1 and whether TGF-β1 itself undergoes palmitoylation remain to be further explored.

#### 4.1.1. Diabetic Kidney Disease (DKD)

DKD occurs in 30–40% of patients with diabetes mellitus (DM). It is a severe complication characterized primarily by renal damage and represents a leading global cause of kidney failure [82]. Early-stage DKD is often asymptomatic. However, with a prolonged duration of diabetes, patients progressively develop symptoms such as a reduced glomerular filtration rate (GFR), proteinuria, and gradual deterioration of renal function. This cascade of pathological changes can further progress to glomerulosclerosis and renal fibrosis, ultimately culminating in end-stage renal disease (ESRD), which is among the primary causes of mortality in diabetes patients [83].

DKD is a common and serious complication of diabetes. Some studies suggest that palmitoylation may be involved in the pathogenesis of DKD. Feng et al. demonstrated a significant increase in palmitoylation levels in the renal tissues of diabetic mice, which was closely linked to the progression of renal fibrosis. The exposure of renal tubular epithelial cells to carboxymethyllysine (CML), a key component of advanced glycation end products (AGEs), increased CD36 palmitoylation. This elevation coincided with the induction of EMT, as evidenced by upregulated α-SMA and vimentin expression and downregulated E-cadherin expression. These findings indicate that palmitoylation is involved in the EMT process of renal tubular epithelial cells during diabetic nephropathy and subsequently leads to the onset of renal fibrosis. Moreover, ZDHHC6 acts as a key regulator in the pathogenesis of DKD by regulating cytoskeletal stability, oxidative stress, and mitochondrial function through podocyte-specific palmitoylation. The loss of ZDHHC6 function leads to podocyte injury and subsequent disruption of the glomerular filtration barrier [71]. These studies indicate that either the inhibition of CD36 palmitoylation or the maintenance of ZDHHC6 function can effectively alleviate the progression of DKD. However, whether CD36 palmitoylation affects blood glucose levels remains to be investigated.

#### 4.1.2. Autosomal-Dominant Polycystic Kidney Disease (ADPKD)

ADPKD is an autosomal-dominant disorder and the fourth most common cause of ESRD [84]. Its underlying cause lies in mutations in the PKD1 and PKD2 genes, which encode PC1 and PC2, respectively [85]. Accumulating evidence has demonstrated that the rate and severity of renal cyst progression in ADPKD are closely correlated with the expression level of functional PC1 on the primary cilium. Consequently, modulation of the trafficking of PC1 to the cilium may represent a tunable therapeutic strategy for ADPKD. Roy et al. revealed that PC1 undergoes palmitoylation at its carboxyl terminus, whereas PC2 does not. In ADPKD, regulation of the palmitoylation at the carboxyl terminus of the PC1 can affect its expression level and trafficking to primary cilia, and thus effectively inhibit the progression of ADPKD. Therefore, the promotion of PC1 palmitoylation could be a new strategy to increase PC1 levels within cilia and potentially intervene in related disease progression [31].

### 4.2. Renal Cell Carcinoma (RCC)

RCC is a prevalent malignant tumor that originates from renal cortical epithelial cells [86] and accounts for approximately 2% of global cancer diagnoses and cancer-related deaths [87]. ccRCC represents the most common subtype and comprises approximately 75–80% of RCC cases [88]. DHHC family-mediated palmitoylation has been demonstrated to regulate protein membrane localization in human cancer cells. Sun et al. elucidated the critical role of ZDHHC2 in ccRCC and revealed that ZDHHC2 stimulates the AKT-mTOR signaling cascade in ccRCC cells by modulating palmitoylation at AGK Cys72. Consequently, this process affects cell proliferation, EMT, and angiogenesis and modulates sensitivity to sunitinib treatment. These conclusions provide a theoretical foundation for targeting ZDHHC2 to enhance the antitumor efficacy of sunitinib in ccRCC [19].

**Figure 6:**
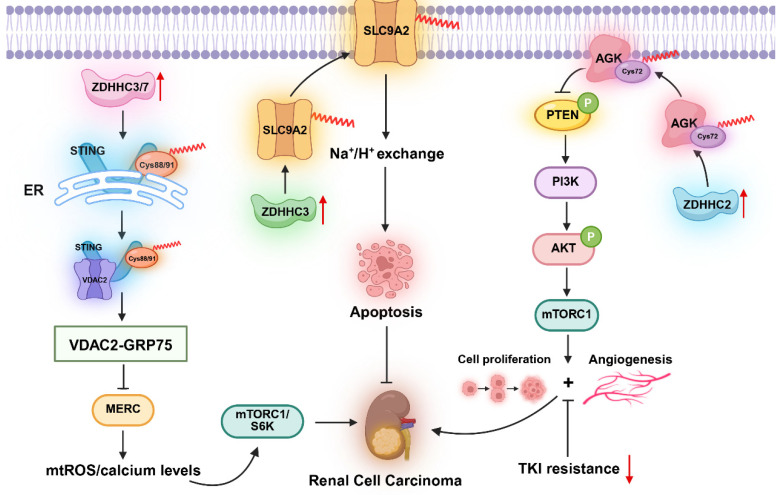
ZDHHC family enzymes regulate the development of renal cell carcinoma through the palmitoylation of STING, SLC9A2, and AGK proteins. The upward red arrow represents the upregulation of protein expression, the downward red arrow represents the downregulation of protein expression.

Furthermore, pharmacological inhibition or genetic deletion of ZDHHC palmitoyltransferases significantly disrupts the STING-VDAC2 interaction and effectively suppresses RCC cell growth [32]. Another study revealed that ZDHHC3 suppresses SLC9A2 expression and impedes S-palmitoylation, and this action inhibits apoptosis in ccRCC cells [18] (Figure 6). Additionally, circRNA sodium channel epithelial 1 subunit alpha (CircSCNN1A) has been identified as a tumor suppressor in RCC. It acts via the miR-421/MPP7 axis to effectively suppress the malignant progression of RCC [89]. Concurrently, bioinformatic analyses have revealed that poor prognosis in patients with ccRCC is associated with low expression levels of ZDHHC7, ZDHHC20, and ZDHHC21 [90]. These findings collectively suggest novel therapeutic avenues for treating ccRCC. Notably, the aberrant expression of ZDHHC genes is closely linked to the initiation and progression of ccRCC. The expression levels of certain ZDHHC genes decrease as the pathological stage advances in patients with ccRCC, which suggests their potential involvement in tumor progression [18]. Cheng et al. further reported that membrane palmitoylated protein 7 (MPP7) is strongly associated with ccRCC prognosis and is a potential biomarker and therapeutic target for this disease [91]. These prediction results based on data correlation provide potential novel targets for the treatment of ccRCC; however, their specific regulatory mechanisms and therapeutic effects remain to be further verified by experiments. Collectively, these discoveries expand beyond elucidating the biological roles of protein palmitoylation in tumor biology to pinpoint novel potential diagnostic markers and therapeutic targets for RCC.

In summary, palmitoylation plays a key regulatory role in the pathogenesis and therapeutic strategies of multiple kidney diseases. The targeting of the palmitoylation, through the regulation of specific palmitoyltransferases and depalmitoyltransferases can substantially influence disease course, yet its therapeutic effects differ according to target proteins and diseases: in renal fibrosis, enhanced palmitoylation of β-catenin or HRAS often exerts anti-effects, whereas in kidney cancers, the inhibition of the palmitoylation of AGK, STING and so on suppresses tumor growth. In addition, palmitoylation has been shown to activate signaling pathways antagonistic to drug action, which weakens the efficacy of targeted therapies and consequently mediates tumor sunitinib resistance. In Table 3, we summarize the current therapeutic strategies that intervene in the palmitoylation of target proteins.

## 5. Techniques for Detecting Protein Palmitoylation

### 5.1. Acyl-Biotinyl Exchange (ABE)

ABE is a biochemical technique specifically designed for the detection of protein palmitoylation. Its principle involves a chemical displacement reaction in which palmitoyl groups on protein cysteine residues are replaced with a biotin tag, and this replacement makes the enrichment and detection of palmitoylated proteins possible [92].

The key steps of ABE are as follows [89]: (1) sulfhydryl blocking: to prevent nonspecific labeling, nonpalmitoylated free thiol groups are first blocked by treatment with N-ethylmaleimide (NEM). (2) Hydroxylamine cleavage: hydroxylamine (NH_2_OH) is added to selectively cleave the thioester linkages that are formed by palmitoylation. This action releases the palmitoyl groups and exposes the modified cysteine thiols. (3) Biotinylation: the newly exposed thiol groups are labeled via conjugation with biotin-HPDP. (4) Affinity enrichment: biotinylated palmitoylated proteins are captured using streptavidin-coated magnetic beads. Semiquantitative analysis of the effects of palmitoylation on target proteins is then performed using Western blotting.

The advantage of the ABE technique lies in its highly specific cleavage of thioester bonds on cysteine residues mediated by hydroxylamine [93]. This process can selectively cleave modifications linked via thioester bonds without affecting other non-thioester cysteine modifications, such as S-nitrosylation and S-glutathionylation. This specificity allows S-acylation modifications (including palmitoylation) to be accurately distinguished from non-thioester modifications. The ABE technique is applicable in both in vitro and in vivo settings. Although it specifically cleaves thioester bonds, it cannot directly distinguish whether the thioester linkage corresponds to S-palmitoylation or other protein thioester modifications, such as those mediated by long-chain or short-chain fatty acids (e.g., S-myristoylation). Consequently, the presence of these non-palmitoylation thioester modifications in samples may lead to false-positive results in ABE assays targeting palmitoylation.

### 5.2. Acyl-Resin-Assisted Capture (Acyl-RAC)

Acyl-RAC is a chemical proteomics technique designed for the enrichment and analysis of acylated protein modifications, including palmitoylation. Its core principle involves initially blocking all nonmodified free thiol groups in the sample using a thiol-reactive reagent, followed by the specific cleavage of thioester bonds formed by palmitoylation with hydroxylamine. This cleavage exposes a new free thiol group at the modified cysteine residue. These newly exposed thiols are then selectively captured using a thiol-reactive resin, ultimately enabling the enrichment and analysis of S-acylated proteins [94].

Acyl-RAC shares a similar principle with ABE: both utilize hydroxylamine specifically cleaving thioester bonds to expose new thiol groups generated by thioester bond breakage. However, ABE focuses on “exchange”: following hydroxylamine cleavage, the newly exposed thiol groups are first labeled via an exchange reaction with a biotinylation reagent, and subsequently, the biotinylated proteins are specifically enriched through streptavidin affinity chromatography. In contrast, Acyl-RAC emphasizes “capture”: after hydroxylamine cleavage, the newly exposed thiols are covalently captured directly by the thiol-reactive resin [95].

Unlike ABE, Acyl-RAC skips the steps of incubating with biotinylation reagents and performing streptavidin affinity purification. By doing so, the experimental process is simplified. However, because Acyl-RAC depends on the hydroxylamine cleavage step, this step will irreversibly damage the original acyl modifications, causing the loss of dynamic modification details. Additionally, because hydroxylamine cleavage acts only on the thioester bond and does not consider the type of acyl chain attached, acyl-RAC cannot reveal the difference between different kinds of thioester-linked acyl modifications.

### 5.3. Radioactive Labeling Assay

Radioactive labeling assays are biological techniques based on the principle of incorporating radioactive nuclides into compound molecules, which leads to their radioactivity. This enables the tracking of a given compound’s metabolic dynamics and localization within biological systems or the environment using radiation detection. In the context of palmitoylation detection, this technique employs radioisotope-labeled palmitic acid (e.g., [^3^H] palmitic acid or [^14^C] palmitic acid) to metabolically label cells. The labeled palmitic acid is incorporated into proteins through palmitoylation.

The target proteins are subsequently selectively isolated via immunoprecipitation, followed by enrichment and purification of the isolated proteins. Radioactive signal bands on the membrane are then detected using autoradiography [96] (exposure to X-ray film) or fluorescence imaging to allow quantitative or semiquantitative analysis of palmitoylation levels. This approach enables direct, highly sensitive dynamic tracking of protein palmitoylation in living cells. However, its operational complexity and low efficiency stem from stringent radiation shielding, specialized waste disposal protocols, and prolonged exposure times during signal detection. Consequently, the radioactive labeling assay has been gradually replaced by click chemistry-based metabolic labeling. Furthermore, this approach is limited to cultured cells and precludes in vivo applications, as it requires the metabolic incorporation of radioactive tracers or synthetic palmitic acid analogs [97].

### 5.4. Click Chemistry

Click chemistry includes Cu(I)-catalyzed alkyne–azide cycloaddition (CuAAC) in particular. It leverages its exceptional efficiency, high selectivity and bioorthogonality to serve as a pivotal tool for detecting protein palmitoylation. Its core principle involves the use of a metabolic labeling strategy—such as employing alkynyl-functionalized palmitic acid analogs—to introduce an alkyne “chemical handle” into palmitoylated proteins within living cells; subsequently, the CuAAC reaction specifically and covalently conjugates azide-bearing reporter molecules (e.g., fluorophores or biotin) to the alkyne moiety so that it forms a triazole linkage and enables visualization [98]. When fluorophores are employed, the triazole ring directly incorporates the fluorogenic group, and this incorporation permits in situ microscopic observation via fluorescence microscopy; when biotin is used, streptavidin-conjugated enzymes or fluorophores facilitate highly sensitive secondary signal amplification, and this amplification ultimately leads to detection through fluorescence imaging. Furthermore, by combining probes in situ, click chemistry allows palmitoylated proteins to be visualized within their subcellular compartments.

Despite its capacity for the specific labeling of alkynyl-modified palmitoylated proteins while minimizing nonspecific binding, the technique’s dependence on Cu^2+^ catalysis introduces potential cytotoxicity at physiological concentrations, which may disrupt normal cellular metabolic processes. Therefore, experimental designs utilizing click chemistry must rigorously consider and implement strategies to mitigate the potential cytotoxic effects of copper ions.

## 6. Research Strategies for Palmitoylation

**Figure 7:**
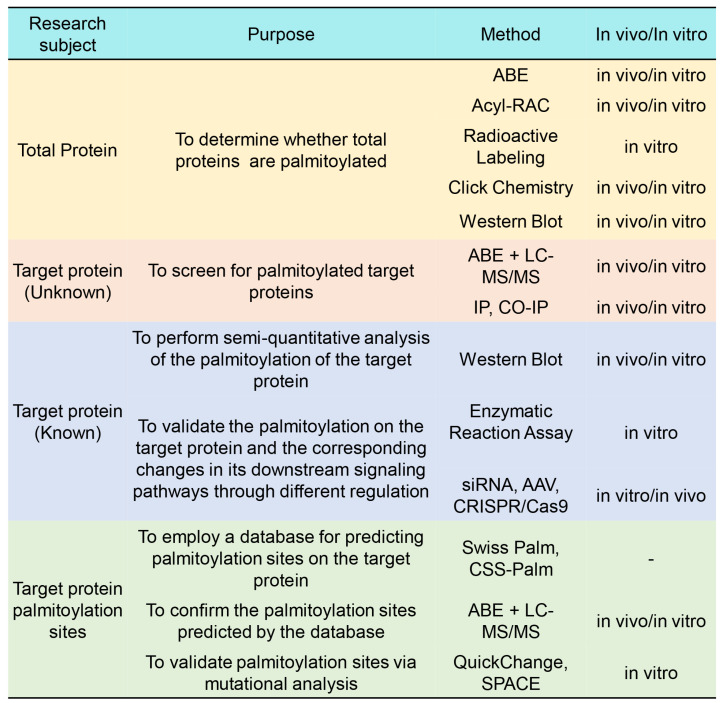
Detection of protein palmitoylation.

Based on previous palmitoylation research, we have summarized a strategy for studying protein palmitoylation for reference (Figure 7).

When the target protein is unknown, ABE, Acyl-RAC, radioactive labeling, click chemistry, and Western blotting can first be used to detect the overall level of palmitoylation in total proteins; subsequently, ABE + LC–MS/MS, IP, and CO-IP can be employed to identify palmitoylated target proteins from the total proteins.

When the target protein is identified, Western blotting can be used to semiquantitatively analyze the palmitoylation level of the target protein, and an enzymatic reaction assay can be employed to validate the activity of palmitoyltransferase-mediated palmitoylation on the target protein. In vitro, knockdown or overexpression of the target protein in relevant cell lines is achieved using adeno-associated virus (AAV), siRNA, or CRISPR-Cas9. In vivo, gene modulation (knockdown or overexpression) of the target protein in specific tissues is performed using AAV or CRISPR-Cas9 in combination with relevant disease models. Alterations in downstream signaling molecules are subsequently detected to evaluate the effects of palmitoylation on the functions of target proteins.

After researchers confirm the palmitoylation of the target protein, they should further reveal its modification sites. Databases such as SwissPalm (https://swisspalm.org/, accessed on 8 February 2026) or CSS-Palm or reference literature and known sites of homologous proteins can be used to predict potential palmitoylated cysteine sites on the target protein; then, the database-predicted palmitoylation sites can be experimentally confirmed using techniques such as ABE or click chemistry combined with LC–MS/MS; additionally, site-directed mutagenesis, such as through QuickChange technology, can be used to mutate the predicted palmitoylated cysteine residue sites to verify the role of the site in palmitoylation modification and its function. In 2020, the team led by J. Keith Joung at Massachusetts General Hospital developed a dual-deaminase base editing system—the Synchronous Programmable Adenine and Cytosine Editor (SPACE) [99]. This system can simultaneously introduce A-to-G and C-to-T base substitutions without opening DNA double strands. This action significantly improves mutation efficiency and accuracy. It is expected to be applied in site-directed mutation research systems in the future and to provide new methods for validating protein palmitoylation modification sites.

## 7. Conclusions

Palmitoylation is recognized as a critical regulatory mechanism in renal physiology and pathology. By covalently attaching palmitic acid to cysteine residues on proteins, palmitoylation alters protein hydrophobicity and local conformation, thereby enabling specific regulation of protein function or localization. Consequently, this mechanism plays a pivotal role in initiating or terminating renal physiological or pathological states. Within the kidney, palmitoylation is extensively involved in physiological processes. It helps maintain water–salt balance, regulate blood pressure, and ensure ciliary function by modulating ion channel activity, preserving receptor function and signal transduction, and influencing protein localization and interactions. Furthermore, palmitoylation plays a critical regulatory role in the pathogenesis of kidney diseases such as renal cell carcinoma, diabetic kidney disease, and polycystic kidney disease. By regulating palmitoylation, palmitoyltransferases can delay the progression of fibrosis, inhibit tumor cell proliferation and promote tumor cell apoptosis. The targeting of specific protein palmitoylation holds promise for the treatment of kidney-related diseases, which provides a basis for the development of specific palmitoylation modulators.

## 8. Future Perspectives

Protein palmitoylation has been preliminarily studied in various kidney diseases, but its role and mechanism in acute kidney injury (AKI) are still unclear. Palmitoylation can influence cell fate. For example, modulation of STING palmitoylation suppresses cancer cell proliferation [32], and inhibition of SLC9A2 palmitoylation reduces apoptosis in renal cell carcinoma [18]. Given the extensive cell death associated with AKI, negatively regulating the palmitoylation of key proteins such as STING and SLC9A2 to inhibit cell death may offer a novel therapeutic strategy to delay AKI.

2-BP is a widely used palmitoylation inhibitor but exhibits off-target effects and cytotoxicity [100]. Given that the inhibitory effect of 2-BP depends on its covalent binding to cysteine residues, and this binding is non-specific, the compound not only covalently binds to and inhibits the active sites of DHHC enzyme but also modifies exposed cysteines on other proteins, such as APT1 and APT2 [101]. Consequently, even if a decrease in the palmitoylation level of a substrate protein is observed, it is impossible to determine which specific DHHC enzyme has been inhibited, since 2-BP simultaneously disrupts the function of multiple DHHC enzymes as well as non-DHHC proteins. Furthermore, due to the aforementioned off-target effects, effective inhibition of palmitoylation generally requires high concentrations of 2-BP and prolonged treatment; however, many cell lines exhibit toxic responses near this working concentration [102]. Therefore, after 2-BP has achieved initial inhibition of substrate protein palmitoylation, validation should be performed through more specific methods, such as mutation of the palmitoylation sites of the substrate protein or the use of siRNA/CRISPR techniques to selectively knock down the DHHC enzymes that catalyze its palmitoylation. Other palmitoylation inhibitors, such as 2-fluoropalmitic acid and cerulenin, also exhibit nonspecific effects [25]. Although the new compound N-cyanomethyl-N-myracrylamide shows improvements in selectivity and toxicity, existing palmitoylation inhibitors generally do not have sufficient specificity [29]. Therefore, the development of highly selective inhibitors that can precisely target specific DHHC enzymes is important.

Advances in new technologies will catalyze research on palmitoylation. For example, researchers can combine single-cell sequencing with palmitoylation omics technology to advance palmitoylation research from the “tissue-average level” to the “single-cell level.” This approach not only identifies specific cell populations that undergo palmitoylation alterations but also enables in-depth analysis of their regulatory networks, thereby reveal the molecular mechanisms of palmitoylation at single-cell resolution. Organoids derived from human stem cells can closely recapitulate the developmental processes and physiological characteristics of human tissues in both structure and function. Thus, it offers an improved model for palmitoylation investigation. With this model, researchers can investigate the dynamic changes in palmitoylation during tissue development, homeostasis, and disease pathogenesis in a context that closely mimics the human body. Based on this foundation, the further integration of organ-on-a-chip models enables high-precision capture of dynamic data on modified proteins in a physiologically relevant environment. This approach offers unique advantages in analyzing the downstream effects of palmitoylation and greatly facilitates a deeper understanding of its functional mechanisms. The application of these new technologies will improve the analysis of the mechanism of palmitoylation in kidney diseases.

## Figures and Tables

**Table 1:** Palmitoylation is involved in the regulation of different proteins with Cys sites in the kidney.

Target Protein	Cys Site	Outcomes	Disease	Cell	Year	References
Xlcaax-1	Cys585, Cys587	Promotes the localization of Xlcaax-1 to the cell membrane	–	XTC cells	1993	[42]
α2AAR	Cys442	Mutations of the α2AAR adrenergic receptor that eliminate detectable palmitoylation do not perturb receptor-G-protein coupling	–	CosM6, MDCK II, LLC-PK1	1993	[43]
CD36	Cys3, Cys7, Cys464, Cys466	Locate the N- and C-terminals on the inner side of the cytoplasmic membrane	–	HEK293	1996	[44]
LH/hCG receptor	Cys621, Cys622	Palmitoylation-deficient mutants are more prone to ligand-induced receptor down-regulation	–	HEK293	1997	[45]
MPR 46	Cys34	Anchor the cytoplasmic domain to the lipid bilayer to prevent the receptor from entering the lysosome for degradation, thereby maintaining its stability and affecting transport and sorting	–	BHK 21	1998	[46]
CD151	Cys11, Cys15, Cys242, Cys243	Regulates the assembly of cell surface tetraspanin–protein interactions	–	A431, MDA-231, HEK293	2002	[47]
B2AR	Cys341	Only the sites were studied, but the downstream effects were not explored	–	HEK293	2005	[48]
RGS7	Cys133	Regulates RGS7 plasma membrane localization	–	HEK293, COS-7	2005	[49]
eNOS	Cys15, Cys26	Regulates the interaction of eNOS with DHHC-PATs and NO release	–	HEK293	2006	[14]
FSHR	Cys629, Cys655	Modification at Cys629 regulates and impairs cell surface expression	–	HEK293	2008	[50]
PMP22	Cys85	Regulates PMP22 epithelial cell shape and movement	–	Schwann, MDCK	2012	[51]
Ankyrin-G	Cys70	S-Palmitoylation of Ankyrin-G is required for function of Ankyrin-G in membrane domain assembly	–	MDCK	2012	[52]
SCR3	Cys160, Cys162, Cys163, Cys165	Affects the efficiency of exosome secretion and subcellular localization	–	HEK293, HeLa SS4	2013	[30]
ABCG1	Cys26, Cys150, Cys311, Cys390, Cys402	Affects ABCG1-mediated cholesterol efflux	–	HEK293, J774A.1	2013	[53]
Barttin	Cys54, Cys56	Regulate the activity of CLC-K channels	–	MDCK II4, HEK293	2015, 2020	[15,54]
D1R	Cys347, Cys351	Regulating its localization in lipid rafts affects cAMP signal transduction, sodium transport in proximal renal tubules, oxidative stress levels and blood pressure homeostasis	–	RPTCs	2020	[25]
Nav β subunits	Cys182	Enhance the binding with lipid rafts and affect their polarization localization and dynamic diffusion on the cell membrane	–	MDCK	2021	[55]
γENaC	Cys33, Cys41	Required for prototypic ENaC activity but are not necessary for amiloride/benzamil-sensitive Na^+^ transport in the distal nephron or colon	–	–	2023	[56]
SERT	Cys147, Cys155	Promotes its cell surface expression and 5-HT uptake capacity	Depression	AD293	2023	[57]
HCN4	Cys93, Cys179	Enhances the magnitude of HCN4-mediated currents	–	HEK293	2023	[58]
β-catenin	Cys300	Regulated by palmitoyltransferase DHHC9 and acyl protein thioesterase APT1 modulates renal fibrosis	CKD	PTCs/HEK293	2023	[23]
STING	Cys88/Cys91	Facilitates VDAC2 binding and maintains RCC growth	RCC	A498	2023	[32]
AGK	Cys72	Promote the translocation of AGK to the plasma membrane and activate the PI3K-AKT-mTOR signaling pathway	ccRCC	A498, 786-O, ACHN	2023	[19]
DGKε	Cys38, Cys40	Fine-tuning its function in different cell compartments by modulating the spatial proximity between the kinase and substrate within the membrane	–	HEK293	2024	[16]

–: indicates none. XTC cell: xenopus tissue culture cell; CosM6: CosM6 is derived from African green monkey kidney cells; MDCK: madin-darby canine kidney; HEK293: human embryonic kidney 293 cells; BHK: baby hamster kidney; A431, MDA-231: primary cell lines derived from human tumors; COS-7 is derived from African green monkey kidney cells; HeLa SS4 (subcloned HeLa cells); J774A.1 is indeed a mouse monocyte/macrophage cell line; RPTCs: renal proximal tubular cells; AD293 is an adherent cell line derived from HEK293; PTCs: primary tubular epithelial cells; A498, 786-O, and ACHN are all derived from human renal cell carcinoma tissue.

**Table 2:** Palmitoylation is involved in the regulation of different proteins in the kidney.

Target Protein	Outcomes	Disease	Cell	Year	References
Xlcaax-1	Promotes Xlcaax-1 protein binding to plasma membrane	–	The epithelial cells in adult kidney distal tubules	1992	[59]
mGIuR	Regulates its function	–	BHK cells	1995	[60]
eNOS	Regulates membrane targeting of other proteins and may also control targeting of eNOS	–	Endothelial cells, Epithelial cells	2003	[61]
BgtR	Affects subunit assembly and functional formation of BgtRs	–	HEK293, PC12	2004	[33]
PSD-95	Disrupts the interaction of PSD-95 with NMDA receptors in cortical neurons and allows NR2A to be cleaved by calpain	–	HEK293	2004	[62]
hδOR	Contributes to the regulation of hδOR Receptor function at the plasma membrane	–	HEK293	2006	[63]
GODZ	Regulates GABAergic innervation in postsynaptic neurons	–	HEK293	2006	[64]
Wnt	Regulates the activity of Wnt1 and Wnt3a and Wnt-induced proliferation in the chick neural tube	–	HEK293	2007	[65]
NaPi-II	Prevents the transporter from delivery to the plasma membrane	–	MDCK	2007	[66]
β_2a_	Interferes with AA CaV1.3b inhibition	–	HEK293	2009	[67]
P2X7R	Controls the P2X7 receptor expression and association with lipid rafts	–	HEK293	2009	[68]
CLIMP-63	Promotes gentamicin-induced dimerization of CLIMP-63	–	COS-7, HEK293, MDCT, NIH-3T3, KPT11, KPT2,KDT3, HEI-OC1, SV-k1	2010	[69]
ankyrin-G, β II-spectrin	Affects the precise assembly of ankyrin-G and βII-spectrin at the lateral membrane of epithelial cells	–	MDCK	2014	[30]
ARL13b	Controls the localization, stability, abundance, and function of ARL13b	–	IMCD3, HEK293	2017	[70]
PC1	Augments PC1 expression in cilia	ADPKD	WT LLC-PK1 cells, LLC-PK1 cells	2018	[31]
CD36	AGES promote the development of renal tubular fine EMT by increasing CD36 palmitoylation level	Diabetic nephropathy renal fibrosis	HK-2	2018	[71,72]
ENaC	Stabilizes interactions of the cytoplasmic terminal regions of the ENaC with the TM domains	–	MDCK, HEK293	2021	[73]
α1AR	DHHC21 contributes to impaired renal perfusion and function during septic injury via promoting α1AR palmitoylation-associated vasoconstriction	Septic	Tubular cell	2021	[21]
TEAD	Affects the formation and stability of YAP/TAZ-TEAD complex	Mesothelioma	NCI-H226, NCI-H2052, NCI-H28, HEK293, ACHN, UO31, SN12C	2024	[40]
Scramb1	Promotes slit diaphragms assembly within lipid raft microdomains	–	garland nephrocytes	2024	[74]

–: indicates none. PC12 cells: pheochromocytoma 12; MDCT: mouse distal convoluted tubule cell line; NIH-3T3: national institutes of health 3T3 cell line; KPT2/11: mouse kidney proximal tubule cell line 2/11; KDT3: mouse kidney distal tubule cell line 3; HEI-OC1: house ear institute-organ of corti 1; SV-k1: stria vascularis-k1 cell line; IMCD3: inner medullary collecting duct cells; LLC-PK1: lilly laboratories culture-porcine kidney cell line; HK-2: human kidney cell line; NCI-H226/H2052/H28: human malignant pleural mesothelioma cell line; ACHN: human renal cell adenocarcinoma cell line; UO31: human renal cancer cell line; SN12C: human renal cell carcinoma cell line.

**Table 3:** Therapeutic avenues that involve palmitoylation in kidney disease.

Therapy	Target	Effect on Palmitoylation and Therapeutic Action	Model	Disease	References
DHHC9 activation (isoniazid); DHHC9 overexpression	β-catenin	Activates or overexpresses DHHC9 to promote β-catenin palmitoylation at Cys300, enhances its ubiquitination and degradation, and inhibits renal fibrosis.	UUO, IRI	Renal fibrosis	[23]
APT1 inhibition (ML348); siRNA APT1	β-catenin	Inhibits or silences APT1 to reduce β-catenin depalmitoylation at Cys300, promotes its ubiquitination and degradation, reduces fibrosis.	UUO, IRI	Renal fibrosis	[23]
ZDHHC18 knockout (Cdh16-Cre)	HRAS	Downregulates ZDHHC18-mediated HRAS palmitoylation, inhibits partial EMT of renal tubular epithelial cells, and alleviates renal fibrosis.	UUO, FA	Renal fibrosis	[20]
HRAS knockout (Cdh16-Cre)	HRAS	Abolishes HRAS palmitoylation via HRAS knockout, inhibits EMT and extracellular matrix deposition, and alleviates renal fibrosis.	UUO	Renal fibrosis	[20]
ZDHHC2 knockout (CRISPR/Cas9)	AGK	Abolishes AGK palmitoylation, blocks the AKT-mTOR pathway, inhibits tumor cell proliferation and angiogenesis, restores tumor sensitivity to sunitinib, and induces apoptosis.	nude mouse xenograft	ccRCC	[19]
AGK knockout (CRISPR/Cas9)	AGK	Knocks out or silences AGK to disrupt downstream signaling of palmitoylation by ZDHHC2 and thereby suppresses tumorigenesis and drug resistance.	nude mouse xenograft	ccRCC	[19]
mTOR inhibitor (Everolimus) *	mTOR	Inhibits mTOR kinase activity to block downstream of the AKT mTOR signaling, reverses ZDHHC2-induced sunitinib resistance, and achieves synergistic anti-tumor effects in mouse models.	nude mouse xenograft	ccRCC	[19]
Palmitoylation inhibitor (2-BP)	STING	Inhibits STING palmitoylation, reduces STING-VDAC2 binding, enhances VDAC2-GRP75 interaction, increases mitochondrial Ca^2+^/ROS, ultimately inhibits tumor cell proliferation and growth.	nude mouse xenograft	ccRCC	[32]
Combination with 2-BP and sorafenib *	STING–VDAC2	Co-inhibits mTORC1/S6K signaling, enhances anti-proliferative and pro-apoptotic effects and jointly suppresses renal cancer cell growth.	nude mouse xenograft	ccRCC	[32]

*: Therapies that indirectly target palmitoylation can enhance or restore the therapeutic efficacy of existing treatments by interfering with palmitoylation-related signaling pathways. UUO: Unilateral Ureteral Obstruction; IRI: Ischemia-Reperfusion Injury; FA: Folic Acid.

## Data Availability

No new data were created or analyzed in this study.
